# Effects of a Short-Term Ballistic Training Program on Performance and Strength Deficit in Elite Youth Female Soccer Players

**DOI:** 10.3390/sports13070237

**Published:** 2025-07-21

**Authors:** Irineu Loturco, Bernardo Requena, Valter P. Mercer, Tulio B. M. A. Moura, Matheus G. A. Alexandre, Lucas D. Tavares, Lucas A. Pereira

**Affiliations:** 1 NAR—Nucleus of High Performance in Sport, São Paulo 04753-060, Brazil; valterpreis@gmail.com (V.P.M.); tuliobernardo@gmail.com (T.B.M.A.M.); lucasa_pereira@outlook.com (L.A.P.); 2 Department of Human Movement Sciences, Federal University of São Paulo, São Paulo 11015-020, Brazil; 3 UCAM Research Center for High Performance Sport, UCAM—Universidad Católica de Murcia, 30107 Murcia, Spain; 4 Facultad de Deporte, UCAM—Universidad Católica de Murcia, 30107 Murcia, Spain; 5 FSI—Football Science Institute, 18016 Granada, Spain; bernardorequena@icloud.com; 6 Scientific Department, São Paulo Football Federation, São Paulo 01141-040, Brazil; matheus.alexandre@fpf.org.br (M.G.A.A.); lucas.tavares@fpf.org.br (L.D.T.)

**Keywords:** team sport, women athletes, football, athletic performance, resistance training, muscle strength

## Abstract

This study examined the effects of a short-term ballistic training program on neuromuscular performance and strength-deficit (SDef) in elite youth female soccer players. Twenty-two under-20 athletes completed a 4-week intervention during the pre-season phase, comprising 12 loaded and 8 unloaded ballistic training sessions performed at maximal intended velocity. Pre- and post-intervention assessments included vertical jumps (squat jump [SJ], countermovement jump [CMJ]), sprinting speed (5, 10, and 20 m), one-repetition maximum (1RM) and peak force (PF) in the half-squat (HS), and peak power and velocity during jump squats (JS) at 30% of 1RM. SDef was calculated as the percentage difference in PF between 1RM in the HS and 30% 1RM. Significant improvements were observed in SJ, CMJ, sprint speed, 1RM-strength, and bar-derived mechanical outputs (ES = 1.18–1.66; *p* < 0.05), with no significant changes in SDef. These results indicate that elite youth female soccer players can improve strength-, power-, and speed-related capacities without compromising force production at higher movement velocities (thus maintaining their SDef). The improvements observed likely reflect the combined effect of a high-frequency, velocity-oriented training approach and a concurrent reduction in traditional technical–tactical (i.e., soccer-specific) training volume. This is the first study to demonstrate that ballistic exercises alone—when properly structured—can enhance neuromuscular performance in female soccer players without increasing SDef. These findings provide practical guidance for practitioners aiming to optimize physical development in team-sport athletes without relying on heavier training loads or extended resistance training sessions—and, especially, without compromising their ability to apply force at higher velocities.

## 1. Introduction

In team sports, optimizing neuromuscular qualities such as strength, power, and speed is essential for enhancing athletic performance [1,2,3]. In this context, the concept of strength deficit (SDef)—defined as the difference between an athlete’s maximal force production capacity and the force applied against lighter loads at higher movement velocities—has recently emerged as a meaningful performance indicator [3,4,5]. While previous studies have investigated the SDef in male populations (e.g., male soccer and rugby players) [5,6], its characterization and responsiveness to training in female athletes, particularly in elite team sports such as soccer, remain unexplored.

Recent evidence in male soccer players suggests that traditional resistance training programs, specifically those relying on moderate to heavy loads (i.e., 60–90% one-repetition maximum [1RM]) [6], tend to increase maximum strength but may also lead to an increase in SDef. These adaptations could potentially impair the production of force under dynamic and high-velocity conditions that define—and are critical to—most sport-specific tasks, especially in soccer (e.g., jumping, sprinting, changing direction, and kicking) [6,7,8]. In contrast, ballistic exercises, performed with light and very-light loads (i.e., using only body mass [BM] as overload), particularly when executed at maximal intended velocity, have been hypothesized to maintain or even reduce SDef while still improving strength- and power-related parameters [4,6,9,10].

To date, no study has directly examined whether it is possible to improve maximum strength, peak force, movement velocity (e.g., jump squat [JS] peak velocity), power, sprint speed, and vertical jump ability without concomitantly increasing SDef in athletic populations. More importantly, no investigation has evaluated this phenomenon (i.e., changes in SDef) in female athletes. Therefore, the present study aimed to analyze the effects of a 4-week strength–power training intervention, comprising only loaded and unloaded ballistic exercises (including plyometric exercises), on a wide range of performance outcomes and SDef in elite youth female soccer players.

Based on previous findings and recommendations [6,11], we hypothesized that it would be possible to enhance strength, power, and sprint performance without an associated increase in SDef. Confirming this hypothesis could have direct implications for training prescription in high-performance sport environments, supporting the implementation of targeted interventions that minimize the unnecessary use of multiple traditional (non-ballistic) exercises and heavy loading intensities, specifically in sports where explosive actions are crucial [10,12,13].

## 2. Materials and Methods

### 2.1. Subjects

Twenty-two elite under-20 female soccer players (age: 17.3 ± 1.7 years; height: 1.65 ± 0.07 m; body mass [BM]: 62.9 ± 8.9 kg), all from the same soccer club and with at least three years of experience in systematic strength–power and plyometric training, participated in this study. These athletes competed in the “São Paulo State Championship”, one of the most important Brazilian under-20 soccer competitions. The study was approved by the local Ethics Committee, and all participants—and, when necessary (i.e., for players under 18 years old), their legal guardians—signed an informed consent form prior to participation.

### 2.2. Study Design

This quasi-experimental study investigated the effects of a ballistic-based resistance training program on strength, power, and speed performance in under-20 female soccer players. Athletes were assessed before and after a 4-week training period, always at the same time of the day, and were properly familiarized with both the training and testing procedures. A detailed overview of the team’s typical weekly training schedule during the intervention is provided in Table 1. Over the course of the training program, players completed 12 loaded ballistic sessions—including JS, jump lunges, and jump deadlifts—and 8 unloaded ballistic sessions that included traditional jumps (i.e., squat jumps (SJ) and countermovement jumps (CMJ), as well as plyometric jumps (i.e., drop jumps and hurdle jumps). Due to time constraints, the height of the boxes (used for drop jumps) and the hurdles was standardized at 30 cm. However, despite the fixed height, players were instructed to jump as high as possible in both jump types, thereby exerting maximal effort in all attempts. The training program was primarily based on previous studies that highlighted the superiority of ballistic JS, especially when performed with light loads, over traditional squats for maximizing force and power output [5,12,13,14], as well as the interference effects (i.e., negative impact) of high volumes of aerobic-based training (e.g., soccer-specific training) on speed–power development [11,15]. Across all training sessions, players were consistently encouraged to perform all exercises at maximal intended velocity. Physical testing followed a fixed sequence: SJ, CMJ, sprinting speed (with split times at 5, 10, and 20 m), 1RM in the half-squat (HS) exercise, and JS at 30% HS 1RM. Prior to testing, players performed a standardized warm-up that included general exercises (e.g., 10 min of moderate-paced running followed by 3 min of dynamic lower-limb stretching) and specific drills (i.e., submaximal attempts of each test).

### 2.3. Procedures

#### 2.3.1. Jump Performance Assessments

Vertical jump performance was evaluated through both the SJ and CMJ protocols. During the SJ, athletes adopted a static position with knees flexed at ≈ 90° for 2 s before jumping vertically, avoiding any preparatory action. For the CMJ, athletes performed a self-selected countermovement followed by full extension of the legs, ensuring natural jumping mechanics were preserved [16]. All trials were conducted with hands placed on the hips. Each athlete performed five trials, with 15 s of rest between attempts. A contact mat (Elite Jump System; S2 Sports, São Paulo, Brazil) was used to record jump height, and the highest jump was retained.

#### 2.3.2. Sprint Speed Testing

Sprint speed was assessed on an indoor track using electronic timing systems. Four sets of photocells (Elite Speed System; S2 Sports, São Paulo, Brazil) were placed at 0, 5, 10, and 20 m along the sprinting track. Athletes performed two maximal-effort sprints from a stationary start, positioned 0.5 m behind the initial set of timing gates. Sprint speed was computed by dividing the distance covered by the time elapsed. A rest period of 5 min was provided between sprints, and the best result was retained.

#### 2.3.3. One-Repetition Maximum in Half-Squat Exercise

Maximum strength was assessed using the HS 1RM test, as previously described [17]. Prior to testing, players completed a standardized warm-up consisting of 5 repetitions at intensities ranging from 40% to 60% of their previously estimated 1RM, based on their most recent 1RM measurements. Three minutes after the warm-up, players were allowed up to 5 trials at approximately 70%, 80%, 90%, and >95% of the estimated 1RM to accurately determine their 1RM [17]. A 3 min rest interval was provided between each successive attempt. In all trials, athletes were instructed to move the barbell as quickly as possible during the concentric phase of the lift. Peak force (PF) was measured during the heaviest load attempts (>95% of the estimated 1RM) using a linear velocity transducer (T-Force Dynamic Measurement System; Ergotech Consulting, Murcia, Spain) sampling at 1000 Hz and attached to the Smith-machine barbell. The 1RM and PF values were normalized to the players’ BM.

#### 2.3.4. Bar-Derived Variables and SDef in the Jump Squat Exercise

Peak power (PP), peak velocity (PV), and PF were assessed during the JS exercise using a load equivalent to 30% of the HS 1RM. The assessment was performed on a Smith Machine (Hammer Strength Equipment, Rosemont, IL, USA). Players completed three maximal-effort repetitions, aiming to move the bar as fast as possible, with 15 s rest intervals between each attempt. A linear velocity transducer (T-Force Dynamic Measurement System; Ergotech Consulting, Murcia, Spain), operating at a sampling frequency of 1000 Hz, was attached to the bar to record mechanical variables. The highest PV value and its corresponding PF and PP values were recorded. All values were normalized by expressing the absolute measurements relative to each athlete’s BM. SDef was calculated as the percentage difference between the PF at 30% 1RM and the PF at 1RM.

### 2.4. Statistical Analysis

Data are presented as means ± standard deviation. Normality was assessed using the Shapiro–Wilk test. Pre- and post-assessment comparisons were conducted using paired *t*-tests. Statistical significance was established at a threshold of *p* < 0.05. Effect sizes (ES) were determined using Cohen’s d [18] and interpreted based on the criteria proposed by Rhea [19] for highly trained individuals: trivial (<0.25), small (0.25–0.50), moderate (0.50–1.00), and large (>1.00). The absolute and relative reliability of the dependent variables was evaluated using the coefficient of variation (CV) and the two-way random intraclass correlation coefficient (ICC), respectively.

## 3. Results

**Figure 1:**
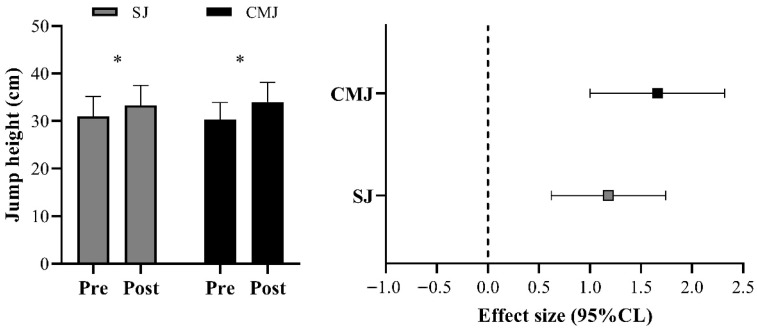
Comparisons of squat jump (SJ) and countermovement jump (CMJ) performance between pre- and post-tests. CL: confidence limits; * *p* < 0.05.

**Figure 2:**
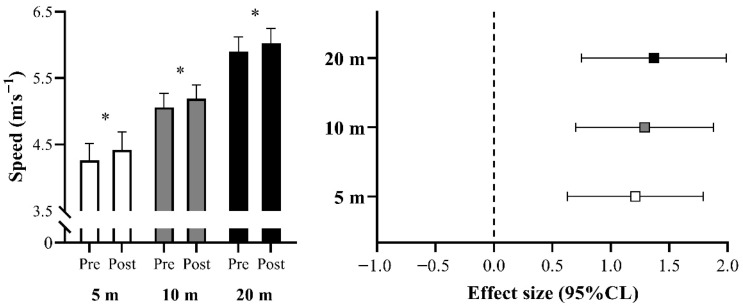
Comparisons of sprinting speed over the different distances tested between pre- and post-tests. CL: confidence limits; * *p* < 0.05.

All variables demonstrated high levels of absolute and relative reliability (i.e., ICC > 0.90 and CV < 10%). Significant increases were observed in SJ and CMJ performance from pre- to post-tests (ES = 1.18 and 1.66, respectively; *p* < 0.05; Figure 1). Table 2 presents the comparisons of PV, PF, and PP measured during the JS exercise with a load corresponding to 30% 1RM, as well as relative strength, PF at 1RM, and SDef at 30% 1RM, between pre- and post-assessments. Figure 2 illustrates the sprint speed comparisons between pre- and post-tests. Significant improvements were detected at all tested distances (ES = 1.21, 1.29, and 1.37 for 5, 10, and 20 m, respectively; *p* < 0.05).

## 4. Discussion

This study is the first to examine the effects of a short-term ballistic training program on a range of speed, power, and strength measures, as well as SDef, in elite youth female soccer players. The results demonstrate that it is indeed possible to simultaneously improve jumping ability (i.e., SJ and CMJ), sprint speed (5, 10, and 20 m), maximum strength, and force–power outputs (JS peak force, velocity, and power), without a concomitant increase in SDef during a highly demanding and congested training period such as the soccer pre-season.

Conducting intervention studies with soccer players during pre-seasons is extremely challenging due to the high volume and specific types of training required to prepare them to cope with competitive demands [6,11,15]. Additionally, the highly traditional training routines followed by coaching staff—characterized by large volumes of soccer-specific training (i.e., technical–tactical sessions) [6,11,15]—and their resistance to major or more innovative training strategies make such studies extremely rare [11]. Even rarer are those reporting consistent and meaningful improvements across a range of performance measures (especially speed and power metrics) within such short time periods (i.e., 3- to 4-week pre-seasons) [15,20,21,22]. Notably, during pre-seasons or other training and competitive phases (e.g., in-season), significant improvements in strength, speed, and power qualities are uncommon not only among female players but also in their male counterparts, especially under elite training conditions [15,20,23,24,25].

In this context, the maintenance of SDef after this 4-week intervention is particularly relevant. Previous evidence in male soccer players has shown that increases in maximum strength following traditional resistance training are frequently accompanied by an increase in SDef, suggesting that athletes tend to utilize lower percentages of their maximum force at higher velocities and against lighter loads, such as their own BM [4,6]. In contrast, the present findings demonstrate that a precise and efficient ballistic training program—emphasizing movement velocity (and lighter loads) rather than heavier loads—can improve neuromechanical performance without impairing force application at higher velocities. These results may reflect the specific design of the training protocol, which prioritized unloaded and light-loaded ballistic exercises (i.e., 20–30% 1RM), along with maximal short sprints (i.e., 10–15 m), performed at high frequency (i.e., three to four strength–power sessions and one to two sprint sessions per week).

An additional factor that likely contributed to the observed improvements in speed and power capabilities in this study was the strategic modification of the soccer-specific training schedule and volume. In close collaboration with the coaching staff, the volume of traditional soccer training (i.e., technical–tactical sessions) was deliberately reduced in favor of higher-intensity, shorter training sessions (i.e., small-sided games lasting to ≈ 30–35 min, divided into 4–5 drills with 4 to 5 min rest intervals between each drill). This adjustment, combined with the high frequency of speed and power training sessions, may be considered an uncommon but apparently effective strategy to adopt during soccer pre-seasons—particularly when the main purpose is to enhance neuromuscular abilities. This innovative and nontraditional training approach potentially improved most strength, speed, and power qualities in the female soccer players, without compromising their ability to apply force at higher velocities (i.e., increasing the SDef).

Although this study did not include a control group—a common limitation in investigations involving competitive athletes [23,26,27,28]—the consistent improvements across multiple neuromechanical variables, supported by prior intra-team seasonal data, provide compelling evidence for the efficacy of the implemented training program [11,15,23]. In previous pre-seasons or short-term studies conducted with team-sport athletes at comparable levels, performance gains—specifically in speed and power—were generally absent or even negative following standard practices characterized by high volumes of sport-specific training [11,15,23]. This marked contrast reinforces the uniqueness and practical relevance of the present findings. However, it is important to note that the current study design does not allow for the isolation of the effects resulting from reduced volumes of soccer-specific training from those associated with high-frequency ballistic training [10,11,15]. Nevertheless, these findings suggest that combining light-loaded (or even unloaded) ballistic exercises with lower volumes of technical–tactical training may be highly effective in enhancing strength, speed, and power performance in elite youth female soccer players.

This study has inherent limitations related to its quasi-experimental design. Due to the applied nature of working with elite youth female soccer players during the pre-season, it was not feasible to include a control group, as splitting the team into separate training conditions would interfere with their preparation for upcoming competitions. Although the absence of a non-training control group limits causal inferences, the significant improvements observed in neuromuscular qualities—particularly sprint speed over different distances—following a short-term ballistic training program represent highly relevant findings, as such improvements are uncommon during short soccer pre-seasons or short training phases [11,15,23,29]. In summary, this study provides valuable and practically important evidence that elite youth female soccer players can improve strength, power, speed, and jump performance without increasing SDef over a 4-week pre-season, provided that the resistance training program is strategically structured and based on ballistic- and velocity-oriented methods [6,10,13]. These results have direct implications for practitioners aiming to optimize neuromuscular capacities in elite sport settings without compromising the transfer of strength and power to sport-specific tasks. Future studies should explore the long-term effects of ballistic training across different phases of the season and compare its impact with other strength–power training strategies. Investigations including male players and employing randomized controlled designs could further support and extend the findings presented here. Additionally, examining individual response patterns may help tailor programs to optimize transfer to sport-specific performance.

## 5. Conclusions

This study provides novel evidence that a short-term, high-frequency ballistic training program comprising both loaded and unloaded exercises performed at maximal intended velocity can improve strength-, power-, and speed-related abilities in elite youth female soccer players without increasing SDef. From an applied perspective, these findings indicate that ballistic training strategies (e.g., JS, jump lunges, and plyometrics) using light or no external loads can be highly effective for enhancing neuromechanical qualities relevant to elite soccer performance, particularly during congested training periods such as the pre-season. When performed under these loaded (or even unloaded) conditions, ballistic exercises can improve force application at higher velocities without compromising an athlete’s ability to produce force against lighter loads (i.e., increasing SDef), potentially supporting better transfer to sport-specific actions (e.g., sprinting, jumping, kicking, and dribbling). Importantly, as previously suggested [11,23], reducing the volume of traditional technical–tactical sessions in favor of shorter, high-intensity drills and speed–power-oriented sessions may optimize physical development without impairing soccer-specific preparation, provided this adjustment is well-integrated with the coaching staff. Coaches working in high-performance female soccer can effectively implement short-term ballistic training schemes to improve athletic performance, without the need for heavier loads or extended training sessions to achieve their objectives. These practical strategies may help overcome traditional barriers to neuromuscular development in elite soccer settings, where time constraints and accumulated fatigue are commonly considered limiting factors.

## Figures and Tables

**Table 1:** Typical weekly training schedule followed by the female soccer team during the 4-week intervention period.

	Monday	Tuesday	Wednesday	Thursday	Friday	Saturday	Sunday
Morning	Rest	Speed warm-up (technical drills followed by 4–6× maximal short sprints of 10–15 m) Technical training	Small-sided games **^#^**	Speed warm-up (technical drills followed by 4–6× maximal short sprints of 10–15 m) Technical training	Small-sided games ^#^	Technical training	Friendly matches ^¥^
Afternoon	* Ballistic training	* Ballistic training	Unloaded jump training ^+^	* Ballistic training	Unloaded jump training ^+^	Rest	Rest

Note: * Resistance exercises included 4–6 sets of 4–6 repetitions of (1) jump squats, (2) alternating jump lunges, and (3) jump deadlifts performed sequentially in this order at ≈ 30% 1RM, with 2–3 min of rest between sets. ^+^ Unloaded jumps consisted of 4–6 sets of 4–6 repetitions of (1) squat jumps, (2) countermovement jumps, (3) drop jumps, and (4) hurdle jumps, performed in this order, with 2–3 min of rest between sets. Regarding the sprint bouts, players started each short sprint from a standing position, with a rest interval of 2–3 min between efforts. Sprints were performed on natural grass (i.e., a soccer field), with players wearing their own soccer shoes. All physical training sessions were performed with the same volume and intensity across the 4-week intervention. **^#^** Small-sided games involved approximately 30–35 min of total playing time, comprising 4–5 drills with 4–5 min of rest between each drill. ^¥^ Two friendly matches were played during the study period, scheduled on alternate Sundays.

**Table 2:** Comparisons of the mechanical variables between pre- and post-intervention assessments.

	Pre	Post	*p*-Value	ES (95% CL)
JS PV at 30% 1RM (m·s^−1^)	1.96 ± 0.12	2.03 ± 0.13	<0.001	1.28 (0.69; 1.86)
JS PF at 30% 1RM (N·kg^−1^)	7.09 ± 0.51	7.42 ± 0.61	0.014	0.57 (0.12; 1.02)
JS PP at 30% 1RM (W·kg^−1^)	11.9 ± 1.4	12.9 ± 1.6	<0.001	0.90 (0.40; 1.39)
1RM rel	1.23 ± 0.13	1.39 ± 0.17	0.032	0.49 (0.04; 0.93)
PF at 1RM (N·kg^−1^)	47.8 ± 11.2	49.5 ± 11.2	0.076	0.40 (−0.04; 0.83)
SDef at 30% 1RM (%)	83.9 ± 6.3	83.9 ± 5.9	0.442	0.17 (−0.26; 0.59)

Note: JS: jump squat; 1RM: one-repetition maximum; PV: peak velocity; PF: peak force; PP: peak power; SDef: strength deficit; ES: effect size; CL: confidence limits.

## Data Availability

The minimal dataset can be found in Supplementary Materials S1.

## Supplementary Materials

The following supporting information can be downloaded at: https://www.mdpi.com/article/10.3390/sports13070237/s1.
